# Blood Coagulation Induced by Iranian Saw-Scaled Viper (*Echis Carinatus*) Venom: Identification, Purification and Characterization of a Prothrombin Activator

**Published:** 2013-11

**Authors:** Mahdi Babaie, Hossein Salmanizadeh, Hossein Zolfagharian

**Affiliations:** 1 Young Researches and Elites Club, Science and Research Branch, Islamic Azad University, Tehran, Iran; 2 Department of Venomous Animals and Antivenom Production, Razi Vaccine and Serum Research Institute, Karaj, Iran

**Keywords:** Blood coagulation, Chromatography, Iranian Echis carinatus, Prothrombin time, Protrombin activator

## Abstract

***Objective(s):*** *Echis carinatus* is one of the venomous snakes in Iran. The venom of Iranian *Echis carinatus* is a rich source of protein with various factors affecting the plasma protein and blood coagulation factor. Some of these proteins exhibit types of enzymatic activities. However, other items are proteins with no enzymatic activity.

***Materials and Methods:*** In order to study the mechanism and effect of the venom on human plasma proteins, the present study has evaluated the effect of crude venom and all fractions. A procoagulant factor (prothrombin activator) was isolated from the venom of the Iranian snake *Echis carinatus* with a combination of gel filtration (Sephadex G-75), ion-exchange chromatography (DEAE- Sepharose) and reverse phase HPLC. Furthermore, proteolytic activity of the crude venom and all fractions on blood coagulation factors such as prothrombin time (PT) was studied.

***Results:*** In the present study, the PT test was reduced from 13.4 s to 8.6 s when human plasma was treated with crude venom (concentraion of venom was 1 mg/ml). The purified procoagulant factor revealed a single protein band in SDS polyacrylamide electrophoresis under reducing conditions and its molecular weight was estimated at about 65 kDa. A single-band protein showed fragment patterns similar to those generated by the group A prothrombin activators, which convert prothrombin into meizothrombin independent of the prothrombinase complex.

***Conclusion:*** This study showed that the fraction which separated from Iranian snake *Echis carinatus* venom can be a prothrombin activators. It can be concluded that this fraction is a procoagulant factor.

## Introduction

Snake venom, a complex mixture principally composed of proteins and peptides, exhibits diverse biological activities that affect several vital systems (1). 

*Echis carinatus* (Saw scaled viper) is a venomous snake found in the desert regions of Iran. The venom of *E. carinatus*, a member of the Viperidae family, affects blood coagulation due to hemostatically active enzymes with procoagulant and anticoagulant activity (2, 3).

The venom of *E. carinatus *affects the blood circulation. This venom is very toxic causing severe tissue and organ damage. The venom of *E. carinatus* is rich in proteins and peptides effective on the hemostatic system, i.e., its acts against some types of factors involving coagulation and fibrinolysis (4, 5). 

*E. carinatus *snake venom especially contains proteins affecting the transformation of the prothrombin into thrombin (6). Prothrombin is the protein which is broken in plasma by ecarin. In fact, this protein cleaves the bond in prothrombin and produces meizothrombin, which is converted into α-thrombin by autolysis (7).

The conversion of the prothrombin into thrombin is one of the central reactions of blood coagulation (8, 9). The physiological activation of prothrombin to the serine proteinase α-thrombin is catalysed by prothrombinase complex consisting of the serine proteinase, factor Xa, cofactor Va and Ca^2+^. Membranes containing anionic phospholipids are essential for the optimal function of this enzyme complex (10, 11). However, the rate of activation is five orders of magnitude lower than the activation by prothrombinase complex (12), and the mechanism of cleavage proceeds through prethrombin-2 rather than through meizothrombin (13).

The venom of Viperidae presents a high level of haemorrhagic, coagulant and proteolytic activities (14). Proteins effective on blood coagulation and existing in the snake venom are classified based on their ability to lengthen or shorten the clotting process into coagulation and anticoagulation proteins (15).

The aim of the present investigation was to study the purification and characterization of porothrombin activator (procoagulant factor) from the Iranian *E. carinatus* venom and to evaluate the procoagulant activity on *in vitro* human plasma.

## Materials and Methods

***Material***

The lyophilized *E. carinatus* venom was obtained from the Department of Venomous Animals and Antivenom Production, Razi Vaccine and Serum Research Institute Karaj, Iran. Sephadex G-75, DEAE-Sepharose, and C18 columns were purchased from the Pharmacia company (Sweden). CaCl_2_ and PT kits were purchased from the Fisher Diagnostics (USA). Protein markers were obtained from BioRad (Hercules, USA). Other reagents and chemicals were of analytical grade from Fluka and Merck.

***Methods***

***Blood collection***

Normal plasma from 20 healthy donors without any history of bleeding or thrombosis was collected from a private clinical laboratory. The citrated blood was centrifuged for 15 min at 3,000 rpm, to get clear plasma. Finally, the PT was estimated.

***Protein determination***

The total protein of crude venom of *E. carinatus*, and its fractions were determined by Lowry method (16).

***Purification and isolation of prothrombin activator***

Purification of the prothrombin activator was performed in three steps. Lyophilized crude venom of *E. carinatus* (50 mg) was dissolved in 4 ml of starting buffer (20 mM ammonium acetate, pH 6.8) and centrifuged at 3,000 rpm for 15 min, 4°C. The supernatant was filtered on a 0.45 microfilter to remove all insoluble materials. The supernatant was then applied into a Superdex G-75 column and eluted with the same buffer. (150 × 3 cm). Fractions were collected at 4°C and their absorbances were recorded at 280 nm. The fractions with proguaolant activity were pooled, lyophilized and dialyzed against 50 mM Tris-HCl, pH 8.2 buffers. The dialyzed sample was centrifuged at 3000 rpm to clear the precipitated proteins. For further purification, the supernatant was loaded into ion exchange column (DEAE-Sepharose) and equilibrated with 50 mM Tris-HCl buffer, pH 8.2 and eluted with a liner gradient of Nacl concentration from 0.0 to 0.5 mM. The fractions exhibiting proguaolant activity in the previous step were pooled and dialyzed overnight at 4°C and applied on HPLC column, C18 (H_2_O, 0.1% trifluoroacetic acid), and eluted with a concentration gradient of solvent B (acetonitrile, 0.1% trifluoroacetic acid) from 0 to 100%, at a flow rate of 0.3 ml/min during 55 min. The peaks were monitored at 280 nm (17).

***Determination of molecular weights***

Electrophoresis on 12/5% polyacrylamide gel was performed according to the method of Laemmli (18). Samples of the crude venom and its fractions were lauded and the molecular weights of protein were determined under reduced conditions. 

***Prothrombin time assay***

For the PT test, 200 μl of the PT reagent was added to 100 μl of citrated plasma (incubated for 1 min at 37°C). The time from the plasma-reagent mixing to the clot formation was defined as the PT and clotting time was recorded (19). The PT test was performed for different concentrations of crude venom and its fractions.

***Coagulant activity***

Normal plasma comprised mixed samples from 20 healthy donors. It was briefly incubated at 37°C and sample aliquots containing some concentration of coagulant fractions or subfraction (50 µg/ml) were added, mixed and shaken and PT was then recorded.

## Results

The present study showed that the crude venom of *E. carinatus* can accelerate the blood coagulation pathway. Our results indicated that as the concentration of venom increases, the PT of plasma decreased (Table 1).

According to the Table 1, when the concentrations of venom increased from 0.01 to 1 mg/ml, the clotting time of plasma reduced from 13.4 to 8.6 sec.

**Table 1:** PT value for different concentration of *E. carinatus* crude venom

Concentrate of venom (mg/ml)	Average of PT (S) *	Preamble
0.01	21 (*P* < 0.001)	Clot is tiny
0.1	12.25 (*P* < 0.005)	increased clot size
1	8.6 (*P* < 0.001)	Clot complete
Control	13.4 (*P* < 0.005)	Clot complete

*n = 8

Total protein of the venom = 48300 μg/ml.

Control = 100 μl of citrated plasma + 200 μl of the PT reagent + Normal saline (Instead of venom).

Test = 100 μl of citrated plasma + 200 μl of the PT reagent + different concentrate of venom.

**Figure 1:**
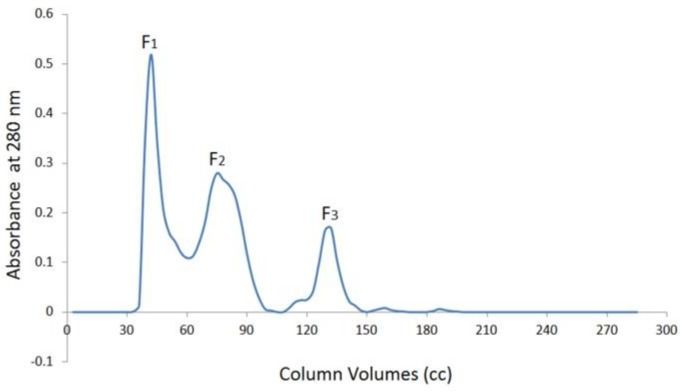
Purification of crude venom of *Echis carinatus *by Sephadex G-75

**Table 2:** PT value for fractions of I*Ec* crude venom

Fractions	PT *
F_1_	12.3 sec
F_2_	35.5 sec
F_3_	More than 300 sec

* n=4, normal PT=13.4, F_1 _(*P* < 0.05) and F_2_ (*P* < 0.01)

PT: prothrombin time

**Figure 2:**
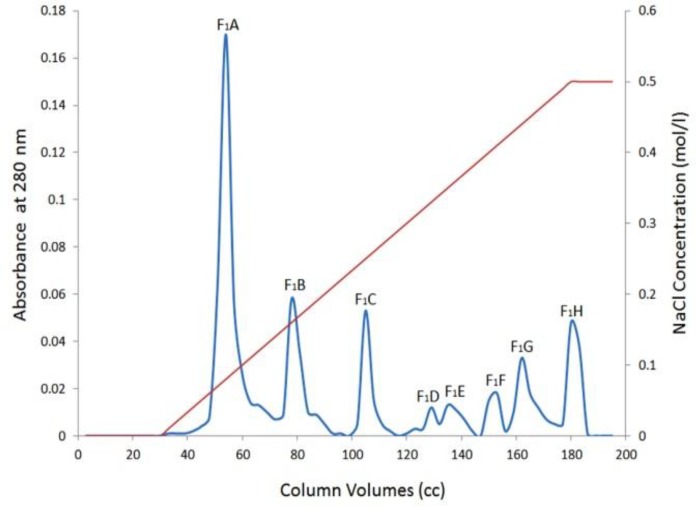
Purification of F_1_ by DEAE-Sepharose chromatography

**Figure 3:**
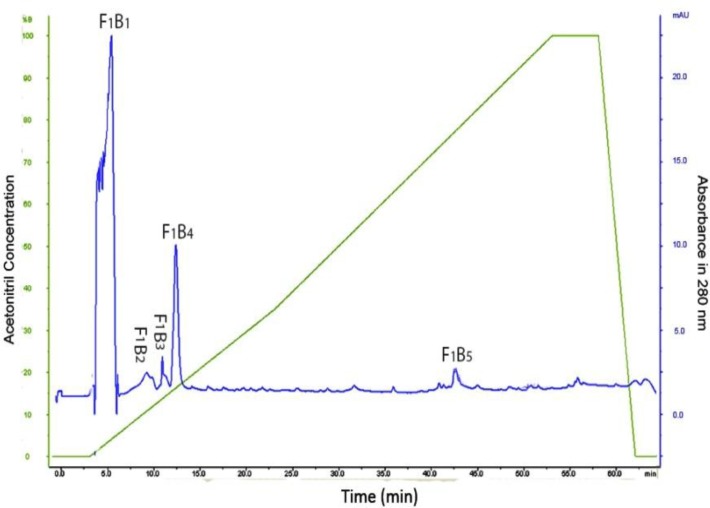
HPLC of F_1_B fraction obtained from DEAE-Sepharose chromatography

**Table 3:** PT value for sub-fractions of *E. carinatus* venom

Fractions	PT *
Fraction F_1_A	14 sec
Fraction F_1_B	8 sec
Fraction F_1_C	70 sec
Fraction F_1_D	52 sec
Fraction F_1_E	90 sec
Fraction F_1_F	56 sec
Fraction F_1_G	More than 300 sec
Fraction F_1_H	95 sec

* n=4, F_1_B (*P*-Value < 0.05)

PT: prothrombin time

**Table 4:** Prothrombin Time for fractions obtained from HPLC

Fractions	Average of PT *
Fraction F_1_B_1_	More than 300 sec
Fraction F_1_B_2_	More than 300 sec
Fraction F_1_B_3_	More than 300 sec
Fraction F_1_B_4_	3 sec
Fraction F_1_B_5_	More than 300 sec

* n=4, F_1_B_4 _(*P*-Value < 0.05) PT: prothrombin time

**Table 5:** Summerized PT value and total protein (crude venom, F_1_, F_1_B, F_1_B_4_)

Step	Protein
Venom	48.3 mg/ml
F_1_	387.77 µg/ml
F_1_B	130 µg/ml
F_1_B_4_	26 µg/ml

PT: prothrombin time

According to the Table 1, when the concentrations of venom increased from 0.01 to 1 mg/ml, the clotting time of plasma reduced from 13.4 to 8.6 sec.

***Purification, isolation and characterization of prothrombin activator***

As it is shown in the Figure 1, the three fractions (F_1_ to F_3_) were obtained By Sephadex G-75. Prothrombin time value was estimated for all the fractions. Our observation showed that the PT value for F_1_ is less than other fractions and this fraction can be considered as a procoagulant factor (Table 2).

Further purification was performed by ion exchange chromatography DEAE-Sepharose. In this step, eight fractions were separated from F_1_A to F_1_H (Figure 2), out of eight fractions, only F_1_B showed procoagulant activity (Table 3).

The F_1_B was pooled, dialyzed and applied to a C18 reversed-phase HPLC column. Our results revealed that five peaks from F_1_B_1_ to F_1_B_5_ were isolated (Figure 3) and out of five fractions, only F_1_B_4_ showed coagulant activity (Table 4). 

Our results summarized in the Table 5, which showed that the PT value significantly decreased in the F_1_B_4_ as compared with PT value of the crude venom. 

**Figure 4:**
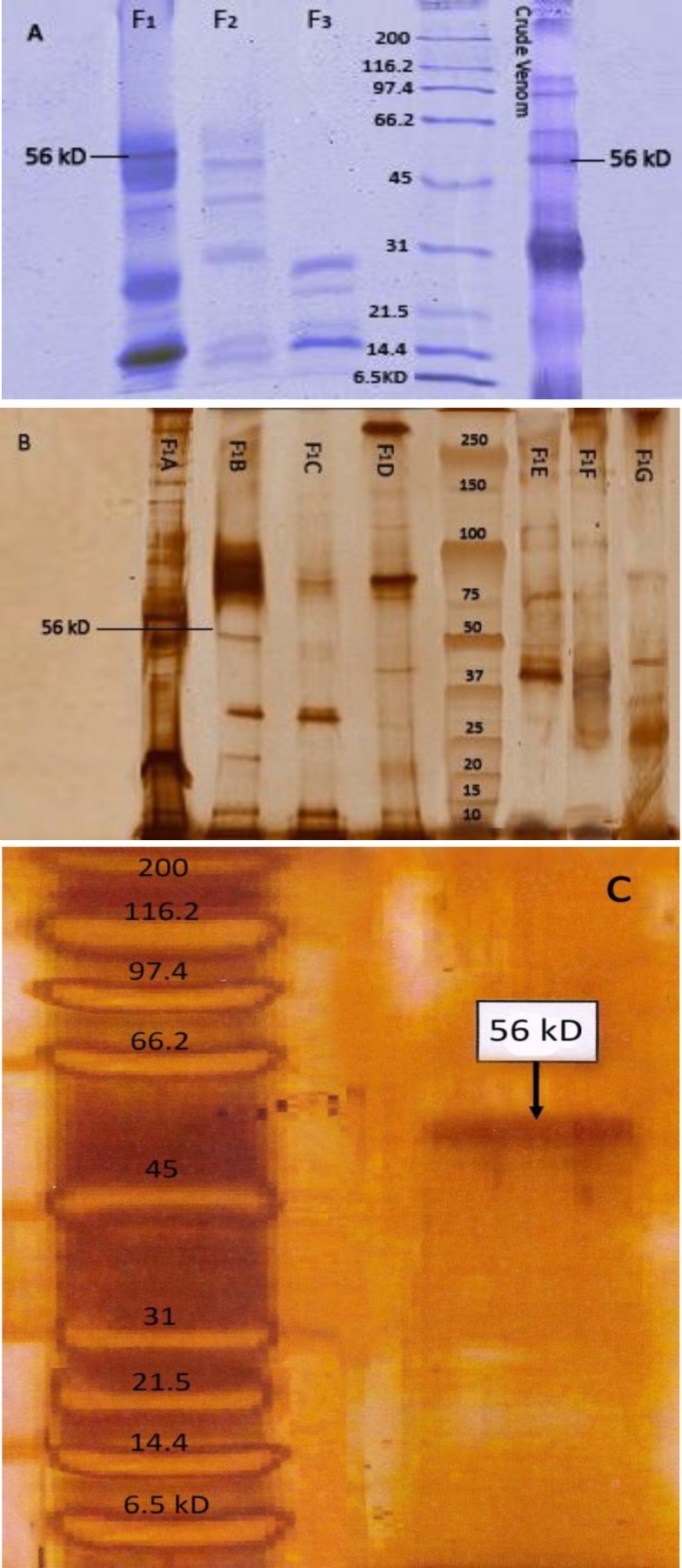
SDS-PAGE pattern of crude venom and its fraction: **A:** Crude venom and its fractions; **B:** Subfractions of F_1__;_ **C:** Fraction of F_1_B_4_

***Purity and determination of molecular weight***

Crude venom and all fractions were analyzed by SDS-PAGE. As it is shown in the SDS-PAGE pattern, the molecular weight of crude venom and all of the fractions were estimated (Figure 4A, 4B and 4C). The molecular weights from the snake venom ranged from 6.5 to 250 kDa and the molecular weight of procoagulant factor was approximately 56 kDa. According to the Figure 4C, a single band of F_1_B_4_ indicates the purity of this protein. 

## Discussion

This study has investigated the venom of Iranian *E. carinatus* which contains a strong procoagulant factor enabling to activate the prothrombin. The functional properties of the *E. carinatus* prothrombin activator are similar to ecarin, the first prothrombin activator which was recently discovered to be present in the venom from *E. carinatus *(20). The venoms of many *E. *species are able to convert prothrombin into thrombin, either directly or indirectly (21).

Under *in vitro* conditions, this venom also displays coagulation properties and increases the blood coagulation cascade. Crude venom from the Iranian snake *E. carinatus *was selected and assayed with PT test. Our results indicated that the Iranian *E. carinatus *venom has a procogulant activity and is able to coagulate human plasma rapidly (Table 1), therefore it may be concluded that I*Ec *venom contains procoagulant factors.

The present study reports an efficient and simple procedure for purification and isolation of procoagulant factor from I*Ec* venom. The fraction F_1_B_4_ was isolated from I*EC* venom by a combination of several methods. Our results revealed that three peaks from F_1_B_1_ to F_1_B_5_ were isolated. In addition, out of five fractions, only F_1_B_4_ showed coagulant activity (Table 4). The molecular weight of this purified fraction was approximately estimated to be 56 kDa (Figure 4C). Our observation showed that the molecular weight of F_1_B_4_ is similar to prothrombin activator enzymes which have been already reported (20). Therefore, this coagulant factor may belong to the intermediate-molecular-weight group of these factors.

By performing the prothrombin Time test on human plasma, the blood coagulation time on fraction F_1_ showed the least coagulation time and fraction F_3_ displayed the highest coagulation time. The total protein of crude venom is 48.3 mg/ml and the PT value is 8.6 s but in the hyper purified fraction with reducing amount of total protein (26.0 µg/ml), the PT value (3 s) also significantly decreased. It may be suggested that with low amount of total protein the PT value decreases.

 Some procoagulant factors, along with its molecular weights, have been reported by Howes JM *et al* in addition to the effects of three novel metalloproteinases (weighting 56 kDa) from the venom of the West African saw-scaled viper, *E. ocellatus* on blood coagulation and platelets (22). Daisuke Yamada *et al* isolated and characterized the carinactivase, a novel prothrombin activator from *E. carinatus *Venom with 62 kDa (23).

Mikarin is the first group of IA prothrombin activator identified in the venom of a viperidae snake. In the case of prothrombin activator, it exhibited prothrombin activation, which was similar to the other group IA prothrombin activators, such as ecarin from *E. Carinatus* (24), aharin from *Agkistrodon halys pallas* (25) and prothrombin activator from *Bothrops atrox* (26).

Over the past 20 years, many metalloproteinase have been isolated from snake venom with a wide variety of biological activities, including hemorrhagic (27), fibrinogenolytic and antiplatelet effects (28), as well as activation of prothrombin and factor X (29).

## Conclusion

Protein with coagulation activities was purified from the venom of *E. carinatus*. The venom of *E. carinatus* including the Iranian *E. carinatus* is one of the coagulation venoms whose function is a pseudothromboplastin action. However, under *in vitro* conditions, this venom will generate high coagulation which is due to activation of the prothrombin. 

It is suggested that, this venom containing procoagulant factors with molecular weight of about 56 kDa. It seems the fraction F_1_B_4_ isolated from I*Ec* to be like ecarin which is already reported. 

## References

[1] Gnanathasan A, Rodrigo C, Peranantharajah T, Coonghe A (2012). Saw-scaled viper bites in Sri Lanka: is it a different subspecies. Clinical evidence from an authenticated case series. Am J Trop Med Hyg.

[2] Matsui T, Fujimura Y, Titani K (2000). Snake venom proteases affecting hemostasis and thrombosis. Biochim Biophys Acta.

[3] Roberto G, Alessio C, Nnadozie S, Hope O, Emiliano F, Helena C (2010). In vitro effects of Echis carinatus venom on the human plasma proteome. Proteomics.

[4] Kularatne SAM, Sivansuthan S, Medagedara SC, Maduwage K, de Silva A ( 2011). Revisiting saw-scaled viper (Echis carinatus) bites in the Jaffna Peninsula of Sri Lanka: distribution, epidemiology and clinical manifestations. Trans R Soc Trop Med Hyg.

[5] Fonseka CL, Jeevagan V, Gnanathasan CA (2013). Life threatening intracerebral haemorrhage following saw-scaled viper (Echis carinatus) envenoming-authenticated case report from Sri Lanka. BMC Emerg Med.

[6] Morita T, Iwanga S (1978). Purification and properties of prothrombin activator from the venom of Echis carinatus. J Biochem.

[7] Lövgren A (2013). Recombinant snake venom prothrombin activators. Bioengineered.

[8] Davie EW, Ratnoff OD (1964). Waterfall sequence for intrinsic blood clotting. Science.

[9] Macfarlane RG (1964). An Enzyme Cascade in the Blood Clotting Mechanism, and it’s Function as a Biochemical Amplifier. Nature.

[10] Jackson CM, Nemerson Y (1980). Blood coagulation. Annu Rev Biochem.

[11] Rosing J, Tans G (1988). Meizothrombin, a major product of factor Xa-catalyzed prothrombin activation. Thromb Haemostasis.

[12] Mann KG (1994). The coagulation explosion. Ann NY Acad Sci.

[13] Heldebrant CM, Noyes C, Kingdon HS, Mann KG (1973). The activation of prothrombin. The partial amino acid sequences at the amino terminal of prothrombin and the intermediates of activation. Biochem Biophys Res Commun.

[14] Maruyama M, Kamiguti AS, Tomy SC, Antonio LC, Sugiki M, Mihara H (1992). Prothrombin and factor X activating properties of Bothropserythromelas venom. Ann Trop Med Parasitol.

[15] Casewell NR, Harrison RA, Wüster W, Wagstaff SC (2009). Comparative venom gland transcriptome surveys of the saw-scaled vipers (Viperidae: Echis) reveal substantial intra-family gene diversity and novel venom transcripts. BMC Genom.

[16] Lowry OH, Rosebrough NJ, Farr AL, Randall RJ (1951). Protein measurement with the Folin phenol reagent. J Biol Chem.

[17] Ghorbanpur M, Zare Mirakabadi A, Zokaee F, Zolfagharian H, Rabiei H (2009). Purification and partial characterization of a coagulant serine protease from the venom of the Iranian snake Agkistrodon halys. J Venom Anim Toxins Incl Trop Dis.

[18] Laemmli UK (1970). Cleavage of structural proteins during the assembly of the head of bacteriophage, T4. Nature.

[19] Rizzo F, Papasouliotis K, Crawford E, Dodkin S, Cue S (2008). Measurement of prothrombin time (PT) and activated partial thromboplastin time (APTT) on canine citrated plasma samples following different storage conditions. Res Vet Sci.

[20] Kini RM, Rao VS, Joseph JS (2001). Procoagulant proteins snake venoms. Thromb Haemostasis.

[21] Helene H, Cassian B (1986). Blood coagulation induced by the venom of Bothrops atrox. 1. Identification, purification, and properties of a prothrombin activator. Biochemistry.

[22] Howes JM, Kamiguti AS, Theakston RDG, Wilkinson MC, Laing GD (2005). Effects of three novel metalloproteinases from the venom of the West African saw-scaled viper, Echis ocellatus on blood coagulation and platelets. Biochim Biophys Acta.

[23] Yamada D, Sekiya F, Morita T (1996). Isolation and characterization of carinactivase, a novel prothrombin activator in Echis carinatus venom with a unique catalytic mechanism. J Biol Chem.

[24] Morita T, Iwanaga S, Suzuki T (1976). The mechanism of activation of bovine prothrombin by an activator isolated from Echis carinatus venom and characterization of the new active intermediates. J Biochem.

[25] Zhang Y, Lee WH, Gao R, Xiong YL, Wang WY, Zhu SW (1998). Effects of Pallas' viper (Agkistrodon halys pallas) venom on blood coagulation and characterization of a prothrombin activator. Toxicon.

[26] Hofmann H, Bon C (1987). Blood coagulation induced by the venom of Bothrops atrox. 1. Identification, purification and properties of a prothrombin activator. Biochemistry.

[27] Paine MJ, Desmond HP, Theakston RD, Crampton JM (1992). Purification, cloning and molecular characterization of a high molecular weight hemorrhagic metalloprotease, jararhagin from Bothrops jararaca venom. Insights into the disintegrin gene family. J Biol Chem.

[28] Siigur E, Siigur J (1991). Purification and characterization of lebetase, a fibrinolytic enzyme from Vipera lebetina (snake) venom. Biochim Biophys Acta.

[29] Takeya H, Nishida S, Miyata T, Kawada S, Saisaka Y, Morita T (1992). Coagulation factor X activating enzyme from Russell's viper venom (RVV-X). A novel metalloproteinase with disintegrin (platelet aggregation inhibitor)-like and C-type lectin-like domains. Biol Chem.

